# Association between physical health and neurocognition in first-episode schizophrenia

**DOI:** 10.3389/fcogn.2024.1387239

**Published:** 2024-05-02

**Authors:** Luke G. Poole, Andrew A. Ude, Hannah M. Perdue, Jonathon R. Bourque, Amber P. Sarwani, Aman P. Dhruve, Brandon L. Alderman

**Affiliations:** Department of Kinesiology and Health, Rutgers University – New Brunswick, New Brunswick, NJ, United States

**Keywords:** schizophrenia, cognition, aerobic exercise, lateralized readiness potential (LRP), P300

## Abstract

**Introduction:**

Impaired cognition is a core feature of schizophrenia that is evident early in the first episode and is frequently accompanied by compromised physical health. Although physical health confers benefits to cognition, it remains unclear whether physical activity, body mass index (BMI) and cardiorespiratory fitness are associated with neurocognition in first episode schizophrenia patients. The purpose of this study was to examine differences in stimulus categorization and motor response selection processes between first-episode schizophrenia patients compared to age-matched controls and explore associations between physical health and these stages of information processing.

**Methods:**

Fourteen young adult patients receiving care following a first episode of psychosis and a matched sample of nonpsychiatric controls completed a visual oddball task from which the P3 and LRP (lateralized readiness potential) event-related potential (ERP) components were extracted to assess stimulus categorization and response selection processes, respectively. Physical activity, aerobic fitness, and BMI were correlated with ERP measures.

**Results:**

Compared with controls, patients had lower physical activity levels and longer P3 and LRP latencies. Regardless of stimulus probability, patients had reduced accuracy and slower reaction times relative to controls. In patients, marginal associations were found between physical activity and P3 difference waveform amplitude, and BMI was negatively associated with parent P3 waveform amplitude.

**Discussion:**

The present findings suggest that cognitive impairment in first-episode schizophrenia spans both stimulus- and response-related stages of information processing, and may be targeted through physical activity interventions.

## 1 Introduction

Cognitive impairment is a core feature of schizophrenia (Barch and Ceaser, 2012; Kahn and Keefe, 2013; Green and Harvey, 2014) that is evident early in the first episode of psychosis and persists over time (Addington and Addington, 2002; Bora and Pantelis, 2015; McCutcheon et al., 2023). Cognitive and psychomotor deficits in schizophrenia are associated with significant functional impairment (Addington and Addington, 2000; Harvey et al., 2012; Vesterager et al., 2012; Cowman et al., 2021), impacting occupational, social, and economic functioning. Although the study of cognition in schizophrenia has been a major focus of research for decades (e.g., Green et al., 2019), this has not yet resulted in the development of novel and effective treatments. Furthermore, although antipsychotic medications are effective at managing psychotic symptoms, they have varying effects on individual cognitive domains and some (e.g., haloperidol) have no influence on cognition (Baldez et al., 2021). Relative to functional impairment, people with schizophrenia tend to live unhealthy lifestyles (e.g., smoking, dietary imprudence and physical inactivity) (Stubbs et al., 2016; Coustals et al., 2020) and are at increased risk for weight gain and obesity (Manu et al., 2015; Afzal et al., 2021). Although physical health metrics such as physical activity and body mass index (BMI) have been linked to cognitive functioning in healthy and psychiatric populations (Erickson et al., 2015, 2019; Prickett et al., 2015; Dye et al., 2017), including schizophrenia (Guo et al., 2013; Kimhy et al., 2014; Leutwyler et al., 2014; Chen et al., 2016), little research has been conducted to examine interactive relationships between physical and cognitive health in schizophrenia (e.g., Jeste et al., 2011; Firth et al., 2018).

Patients with schizophrenia engage in significantly less moderate-to-vigorous physical activity (Stubbs et al., 2016), have lower aerobic capacity (VO_2_ peak), and higher BMI values relative to non-patients, which predict cognitive and social-occupational impairment (Kimhy et al., 2014). Increasing physical activity through behavioral interventions has been shown to enhance cognitive performance in schizophrenia patients. For example, Firth et al. (2017) conducted a meta-analysis of 10 studies (including 7 RCTs) and found that exercise significantly improved global cognition (*g* = 0.43), working memory (*g* = 0.39), social cognition (*g* = 0.71), and attention (*g* = 0.66) in people with schizophrenia. Cardiovascular risk factors (Hagi et al., 2021), including obesity (McWhinney et al., 2021), have also been shown to be associated with cognitive impairment and accelerated brain aging. Further, Kimhy et al. (2014) found significantly lower aerobic fitness levels and higher BMI in schizophrenia patients relative to age- and gender-matched controls, and both were significantly correlated with cognitive functioning. While these studies suggest that these measures of physical health may serve as treatment targets to benefit cognitive function in schizophrenia, few studies have directly examined their potential impact on psychomotor slowing, which has been referred to as “the closest thing to a North-star” in schizophrenia research (Cancro et al., 1971).

Information processing deficits are a primary contributor to cognitive impairment in schizophrenia, making it a worthwhile target for behavioral and pharmacological interventions. It shows the largest magnitude of impairment in schizophrenia (*g* = −1.57) relative to other key aspects of cognition (e.g., episodic memory: *g* = −1.25 and executive function: *g* = −1.00; Dickinson et al., 2007), and is associated with future functional impairment (Milev et al., 2005; Nuechterlein et al., 2011). Information processing deficits in schizophrenia have historically been evidenced by psychomotor slowing (Frith, 1979; Nuechterlein and Dawson, 1984; Braff, 1993). However, the origins of psychomotor slowing remain relatively unknown due to typically used end-state behavioral measures such as reaction time (e.g., Dickinson et al., 2007; Kalkstein et al., 2010). Although behavioral performance metrics are useful, they obscure the granular understanding of where (or *when*) within the stages of information processing psychomotor slowing begins to occur. Event-related potentials (ERPs) are direct and instantaneous measurements of neural activity time-locked to events (Luck and Kappenman, 2011; Luck, 2014), and can be used to examine specific stages of information processing (e.g., Luck et al., 2009; Kappenman et al., 2012).

Luck et al. (2009) assessed early stimulus-related (i.e., stimulus perception and categorization) and later response-related (i.e., selection and preparation) information processing stages in middle-aged patients (age = ~47 years) with schizophrenia or schizoaffective disorder during a simple stimulus discrimination task that required participants to identify and differentially respond to rare target stimuli and frequent standard stimuli. Early and later stages of information processing were assessed using the P300 (P3) and lateralized readiness potential (LRP) ERP components, respectively. The P3 is a positive-going stimulus-locked ERP component that reflects the updating of mental representations stored within working memory and is maximal between 300 and 700 ms across parietal regions of the scalp (Polich, 2012). The LRP is a negative-going component that reflects motor response selection and preparation processes (Coles, 1989) and is maximal between 200 and 600 ms following stimulus presentation across central regions of the scalp. Using this approach, Luck et al. (2009) found attenuated and delayed LRP components in patients relative to controls despite comparable P3 difference waveforms, suggesting that information processing deficits in adult schizophrenia patients are primarily a consequence of deficits in response selection and preparation rather than earlier perception and categorization processes; it remains unknown whether similar temporal deficits are observed early in the course of the disease, and whether these deficits are associated with metrics of physical health.

Although evidence suggesting that physical health confers benefits to information processing in schizophrenia, it is unknown whether they are associated with temporal deficits in information processing in first episode schizophrenia patients. To this end, the purpose of this study was to examine early and later stages of information processing in young adults with first-episode schizophrenia using the P3 and LRP difference waveforms, and to explore their association with physical activity, cardiorespiratory fitness, and body mass index (BMI). We hypothesized that patients would exhibit slower reaction times and reduced accuracy relative to controls, which would be driven by deficits in motor response selection and preparation processes (Luck et al., 2009; Kappenman et al., 2012). We further hypothesized that greater physical activity (Leutwyler et al., 2014; Chen et al., 2016; Firth et al., 2017), higher aerobic fitness (Kimhy et al., 2014), and lower BMI (Guo et al., 2013; Kimhy et al., 2014) would be associated with faster processing speed and enhanced cognitive performance, as indicated by shorter reaction time and increased accuracy, along with reduced ERP latencies and greater ERP amplitudes.

## 2 Methods

### 2.1 Participants

Adult patients receiving care from Rutgers University Behavioral Health Care (UBHC) between the ages of 18–35 years who recently experienced a first episode of psychosis were recruited for a 12-week trial of aerobic and resistance exercise on neurocognition in schizophrenia. Data in this report are from the pre-intervention baseline assessment prior to any physical training. Patients were eligible if they were within 18–35 years of age, had experienced a first episode of psychosis within the past 2 years, were currently receiving treatment and planned to continue treatment for at least 16 weeks, and had normal or corrected-to-normal vision. Psychiatrically healthy control participants matched for age were selected from a previous cross-sectional study that included measures of aerobic fitness and behavioral and EEG measures identical to those collected in the patient sample (see Brush et al., 2020 for full details). Exclusionary criteria included presence of any history of head injury that resulted in a loss of consciousness lasting 10 or more minutes or the presence of any musculoskeletal or cardiovascular conditions contraindicating exercise participation. Healthy control participants were ineligible based on the same exclusion criteria, in addition to current use of psychoactive medication. Participants provided informed consent and the study protocol was in accordance with ethical guidelines of the Helsinki Declaration and approved by the university's Institutional Review Board.

Twenty patients with schizophrenia met the criteria for inclusion. However, six patients were excluded from analysis due to excessive artifacts, yielding a final sample of 14 patients and an equal number of age-matched healthy controls. Eleven of the 14 patients were receiving antipsychotic medication (Abilify: *n* = 5; Zyprexa: *n* = 2, Olanzapine: *n* = 2; Aripiprazole: *n* = 1, Clozaril: *n* = 1; Invega: *n* = 1; combination of Olanzapine and Aripiprazole: *n* = 1; unspecified: *n* = 1). The duration of antipsychotic use was reported by seven patients, which ranged from 2 to 54 months. One patient reported using antipsychotic medication for 54 months; without this patient included, the mean duration of antipsychotic use was *M* = 6.0, *SD* = 2.3 months (mean duration was *M* = 12.9, *SD* = 18.3 months with the patient included). Five patients were also taking antidepressant medication (Zoloft: *n* = 2; Fluoxetine: *n* = 1, Lexapro: *n* = 1; Luvox: *n* = 1; Wellbutrin: *n* = 1), with one patient receiving two medications (Zoloft and Wellbutrin). In addition to psychotropic medications, one patient was receiving medication for a gastrointestinal condition (Uceris and Mesalamine), another was receiving medication for a respiratory condition (Qvar), and a third was receiving an anticholinergic (Benztropine).

### 2.2 Measures

#### 2.2.1 General health history

Basic sociodemographic and health history information was collected at baseline. A general health history questionnaire was used to assess age, sex, medical history, cardiovascular health and risk factors, past and current medical diagnoses, past surgeries, tobacco and alcohol use, and medication history. Height and weight were measured using a stadiometer and scale (Healthometer 500 KL Fitness Scale, Healthometer Professional, McCook, IL) for the calculation of BMI.

#### 2.2.2 Self-reported physical activity

Current physical activity participation was measured using the International Physical Activity Questionnaire Short Form (IPAQ-SF) (Craig et al., 2003), which is a valid and reliable self-report measure of physical activity and inactivity among adults between the ages of 18–65 years, including adults with schizophrenia (Faulkner et al., 2006; Esan and Ephraim-Oluwanuga, 2022). The IPAQ-SF examines three specific types of activity performed in the preceding 7 days, including categories of low, moderate, and vigorous intensity physical activity. Physical activity was calculated as the total amount of activity performed in the last week (MET-mins/week), calculated by summing the duration (i.e., minutes) and frequency (i.e., days) of walking, moderate-intensity, and vigorous-intensity activities, and multiplying the MET score of an activity by the minutes performed.

#### 2.2.3 Aerobic fitness assessment

Relative peak aerobic fitness (ml/kg/min) was assessed during a graded exercise test on a motor-driven treadmill using a modified Bruce protocol in accordance with American College of Sports Medicine (2017) guidelines. After a five-minute warm-up, the speed and grade of the treadmill was increased every 2 min until volitional exhaustion or >85% age-predicted maximal heart rate (220 bpm—age in years) was reached. Gas exchange was sampled every 15 s throughout the test using a computerized indirect calorimetry system (TrueOne 2400 Metabolic Measurement System; Parvo Medics, Sandy, UT, USA). VO_2_ peak values were determined when at least two of the following criteria were met: (1) a plateau in VO_2_ despite a progressive increase in workload; (2) maximal heart rate within 10 beats per minute (bpm) of age-predicted heart rate maximum; or (3) a respiratory exchange ratio >1.10.

### 2.3 Modified oddball task

A modified visual oddball paradigm was administered using E-Prime Professional version 2.0 software (Psychology Software Tools, Inc., Pittsburgh, PA, USA). Individual stimuli consisted of black letters (A–E) and digits (2–6) displayed on a light gray background. Trials began with a stimulus (letter or digit) displayed for 200 ms followed by a randomly jittered intertrial interval ranging between 1,100 and 1,500 ms. Participants were instructed to fix their gaze on a centered black fixation circle that was continuously visible on the monitor throughout the duration of the task, and to respond as quickly and accurately as possible to the presented target stimulus. In half of the trials, participants responded to letters with one hand and to numbers with the other hand. This was reversed for the second half of the task. Stimuli were presented on a 17 in. (43.18 cm) Dell LCD computer monitor placed ~70 cm from the participant's head at eye level. A Logitech F310 handheld game pad (Logitech, Newark, CA, USA) was used to record subject responses by pressing a trigger with the left or right index finger.

The visual oddball task consisted of six blocks of 80 trials (480 total trials). Participants completed three types of blocks. In the first block type, numeric digits appeared on 80% of the trials (frequent) and letters appeared on 20% of the trials (rare). In the second block type, alphanumeric probabilities were reversed such that letters appeared on 80% of the trials and numbers appeared on 20% of the trials. In the third block type, number and letter presentation were equiprobable (50% for either stimulus type). Participants completed two of each block types, which were randomized in order across subjects. Reaction time and accuracy were extracted from trials answered within 200 to 2,000 ms following stimulus presentations and within 2 SD of median reaction time to produce a more normal distribution. The number of trials removed using this threshold did not differ between groups, *t*_(24)_ = 1.06, *p* = 0.30 (patients: 17.46 ± 4.43 trials; controls: 15.77 ± 3.70 trials). Trial accuracy was transformed from percentage of trials answered correctly to the arcsine of the square root to comply with assumptions of normality (McDonald, 2009).

### 2.4 Electroencephalography measures

#### 2.4.1 EEG recording

EEG data were recorded from a 33-electrode actiCap (Brain Products, GmbH; Munich, Germany) arranged according to the 10–20 international systems. Electrooculogram (EOG) activity was recorded from one electrode placed 2 cm lateral to the outer canthus of the left eye (HEOG) and one electrode placed 2 cm below the right eye (VEOG). Data were recorded using an Electrical Geodesics, Inc. (EGI; Eugene, OR, USA) amplifier system (20,000 gain, bandpass=0.10–100 Hz), and were online referenced to the vertex electrode (Cz). Data were digitized at 500 Hz with a 24-bit analog-to-digital converter and were visualized in NetStation 4.0. Impedance values were kept below 20 kΩ throughout the EEG recording.

#### 2.4.2 EEG data processing

EEG data were exported from NetStation 4.0 to the EEGLAB toolbox version 2022.0.0 (Delorme and Makeig, 2004) in MATLAB version R2022b (Mathworks, Inc., Natick, MA, USA) for data processing. Data were bandpass filtered using a 2nd order infinite impulse response Butterworth filter of 0.1–30 Hz and were adjusted for DC offset. Continuous EEG data were visually inspected to identify and remove any segments containing large muscle-related artifacts or extreme offsets. Data were re-referenced offline to the average of left and right mastoids (TP9, TP10). Independent component analysis (ICA) was conducted to identify and correct common artifacts related to EOG activity. Stimulus-locked epochs with a baseline correction period of −200 to 0 ms prior to stimulus presentation were extracted from ICA-reconstructed EEG data for ERP analysis.

Subtraction-based ERP difference waveforms were used to isolate specific cognitive processes related to early and later attentional processes (see Luck et al., 2009; Luck, 2014). Primary P3 measurements were extracted from the rare-minus-frequent P3 difference waveform, which were collapsed across alphanumeric stimuli type and response hand. The LRP measurement was extracted from a contralateral-minus-ipsilateral waveform relative to the responding hand for a given trial (Smulders et al., 2012). The P3 was measured at the midline parietal electrode site (Pz), and the LRP at lateral central sites (C3/C4) (Luck et al., 2009). Both P3 and LRP measurements were extracted from correct response trials made within 200 to 2,000 ms following stimulus presentation.

Latency and amplitude values were calculated for the stimulus-locked P3 and LRP difference waveform components. P3 difference waveform measures were extracted from a 350–700 ms window. The LRP was isolated from a 200–600 ms window following stimulus presentation (Luck et al., 2009; Kappenman et al., 2012; Brush et al., 2020). Latency was measured as 50% fractional area latency and 50% peak onset latency for the P3 and LRP, respectively (Luck et al., 2009). Fractional area latency for the P3 waveforms was defined as the timepoint that divided the area under the curve across the measurement window into two equal halves (i.e., 50% area under the curve). This measure is analogous to peak latency but affords greater statistical power as well as more straightforward comparisons with median reaction time (Luck et al., 2009). Onset latency for the LRP was defined as the time point at which voltage reached 50% of local peak amplitude within the defined measurement window. This latency metric is used for the LRP component because it continues through response execution, where it becomes contaminated by proprioceptive and tactile feedback (see Luck and Gaspelin, 2017). Amplitudes for both ERP components were measured as the mean amplitude within the given latency window. The parent P3 waveform component was also assessed for comparability to previous studies. Parent P3 component measures were extracted from a slightly wider 300–700 ms window following stimulus presentation (Luck et al., 2009; Brush et al., 2020).

### 2.5 Statistical analysis

Descriptive statistics were calculated as means and standard deviations (SD) for continuous variables and total n and percentages (%) for categorical variables. Between-group comparisons in the health metrics of physical activity, aerobic fitness, and BMI were conducted using independent samples *t*-tests. Independent samples *t*-tests were also used to assess group differences in the amplitude and latency of the P3 and LRP difference waveforms. Test selection was in-line with our expectation that patients relative to healthy controls would exhibit comparable P3 difference waveform amplitude and latency, but blunted LRP amplitude (i.e., closer to 0 μV) and longer latency in patients relative to healthy controls (Luck et al., 2009). Between group effect sizes are reported as Hedges' *g*. Separate 2 × 3 mixed factorial analyses of variance with repeated measures were used to assess the effects of Group (Group: patients, healthy controls), Probability (Probability: frequent, equiprobable, rare), and their interaction on behavioral (reaction time and accuracy) and ERP measures (i.e., using LRP difference waveform and the parent P3 latency and amplitude). In cases of nonsphericity, the Greenhouse-Geisser epsilon correction was used (Jennings and Wood, 1976). Significant main effects and interactions were further explored using one-way ANOVAs and Bonferroni-corrected *post-hoc* pairwise comparisons. Lastly, Pearson correlations were conducted to determine associations between behavioral and ERP measures and between physical health metrics and P3/LRP amplitude and latency measures. Correlations were conducted across the entire sample and separately within groups. As per convention, values were considered statistically significant at *p* < 0.05. Considering the small sample size and for reader information, *p*-values approaching significance (< 0.1) are also shown. All statistical analyses were performed using SPSS version 26.

## 3 Results

### 3.1 Demographics

Participants' demographic and physical health characteristics are shown in Table 1. Patients reported lower amounts of physical activity than controls *t*_(15.61)_ = −3.18, *p* < 0.01, *g* = 1.21, engaging in ~25% of the total physical activity engaged in by their nonpsychiatric counterparts. While patients were less aerobically fit, *t*_(26)_ = −1.64, *g* = −0.60, *p* = 0.11 and had higher BMI values relative to controls, *t*_(26)_ = 1.31, *g* = 0.48, *p* = 0.2, these differences were not significant.

**Table 1:** Participant demographic and physical health data by group.

**Variable**	**Patients**	**Controls**	**Statistics**		
*N* (male)	14 (12)	14 (10)	15.5-27,15.2204.5pt ***t***	***p***	***g***
Age (years)	21.7 (3.5)	23.0 (5.3)	*t*_(26)_ = 0.76	0.45	0.29
BMI (kg/m^2^)	26.9 (4.7)	24.8 (3.6)	*t*_(26)_ = 1.31	0.20	0.48
Physical activity (MET-mins/week)	695.5 (639.3)	2750.6 (2156.8)	*t*_(15.61)_ = −3.18	< 0.01	−1.21
VO_2_ (ml/kg/min)	33.7 (7.7)	38.9 (9.2)	*t*_(26)_ = −1.64	0.11	−0.60

Data are represented as M and (SD); BMI, body mass index.

### 3.2 Behavioral performance

Means and standard deviations for behavioral performance measures are presented in Table 2. There was a difference in total number of trials per group with fewer total trials in patients relative to controls, *t*_(19.19)_ = 2.82, *p* =0.01 *g* = 1.03. This was observed across all trial types, with fewer frequent [*t*_(17.37)_ = 2.53, *p* < 0.05, *g* = 0.93], equiprobable [*t*_(26)_ = 2.40, *p* < 0.05, *g* = 0.88], and rare trials [*t*_(26)_ = 2.09, *p* < 0.05, *g* = 0.77] in patients relative to controls (see Table 3).

**Table 2:** Descriptive data for behavioral performance by group and stimulus probability.

	**Patients**				**Controls**				**Statistics**		
									**Group**	**Probability**	**Group** × **probability**
**Dependent variable**	**Group**	**Freq**	**Rare**	**Equi**	**Group**	**Freq**	**Rare**	**Equi**	**df** = **1,25**	**df** = **2,50**	**df** = **2,50**
Accuracy (%)	91.7 (6.5)	94.8 (7.5)	88.1 (9.9)	92.2 (11.5)	96.8 (6.7)	98.1 (1.4)	96.5 (3.6)	96.0 (3.6)	*F* = 4.26; *p* = 0.05; *ηp^2^* = 0.15	*F* = 10.28; *p* = 0.01; *ηp^2^* = 0.29	*F* = 4.79; *p* = 0.01; *ηp^2^* = 0.16
Median reaction time (ms)	326.22 (34.24)	302.62 (30.37)	345.12 (47.53)	330.92 (35.58)	297.70 (34.24)	281.11 (26.95)	304.1 (34.7)	307.89 (38.5)	*F* = 4.68; *p* = 0.05; *ηp^2^* = 0.16	*F* = 33.14; *p* = 0.001; *ηp^2^* = 0.57	*F* = 3.15; *p* = 0.05; *ηp^2^* = 0.11

Behavioral performance measures are displayed as M and (SD).

**Table 3:** Number of trials by trial type and group.

**Trial type**	**Patients**	**Controls**	***t***	***p***	***g***
Rare	42.36 (11.59)	50.29 (8.24)	*t*_(26)_ = 2.09	< 0.05	−0.77
Equiprobable	125.86 (22.04)	142.64 (14.16)	*t*_(26)_ = 2.40	< 0.05	−0.88
Frequent	207.71 (38.27)	235.79 (15.91)	*t*_(17.37)_ = 2.53	< 0.05	−0.93
Total	375.93 (62.66)	428.71 (31.54)	*t*_(19.92)_ = 2.82	0.01	−1.03

Data are represented as M and (SD).

#### 3.2.1 Accuracy

The 2 x 3 repeated measures ANOVA for accuracy revealed a significant main effect of group, *F*_(1, 25)_ = 4.26, *p* = 0.05, η^2^p = 0.15, and probability, *F*_(2, 50)_ = 10.28, *p* < 0.01, η^2^p = 0.29, and an interaction between these two factors, *F*_(2, 50)_ = 4.79, *p* = 0.01, η^2^p =0.16. The group and probability main effects indicated patients were less accurate relative to healthy controls and lower accuracy was observed for rare and equiprobable trials relative to frequent trials. Exploration of the group x probability interaction revealed lower accuracy for rare trials among patients relative to controls, *F*_(1, 25)_ = 8.38, *p* < 0.01, η^2^ = 0.25, but similar accuracy for frequent, *F*_(1, 25)_ = 2.9, *p* = 0.10, η^2^ = 0.10, and equiprobable trials *F*_(1, 25)_ = 1.18, *p* = 0.29, η^2^ = 0.05.

#### 3.2.2 Reaction time

The 2 x 3 repeated measures ANOVA for reaction time revealed a significant main effect of group, *F*_(1, 25)_ = 4.68, *p* < 0.05, η^2^p = 0.16, and probability, *F*_(2, 50)_ = 33.14, *p* < 0.001, η^2^p = 0.57, and an interaction between these factors, *F*_(2, 50)_ = 3.15, *p* = 0.05, η^2^p = 0.11. Patients had significantly slower reaction times relative to controls and slower reaction times were observed for rare and equiprobable trials relative to frequent trials. The significant interaction revealed slower reaction times for rare stimuli among patients relative to controls, *F*_(1, 25)_ = 6.62, *p* < 0.05, η^2^ = 0.21 and marginally significant slower frequent trial reaction times for patients relative to controls, *F*_(1, 25)_ = 3.80, *p* = 0.06, η^2^ = 0.13.

### 3.3 ERP analyses

**Figure 1:**
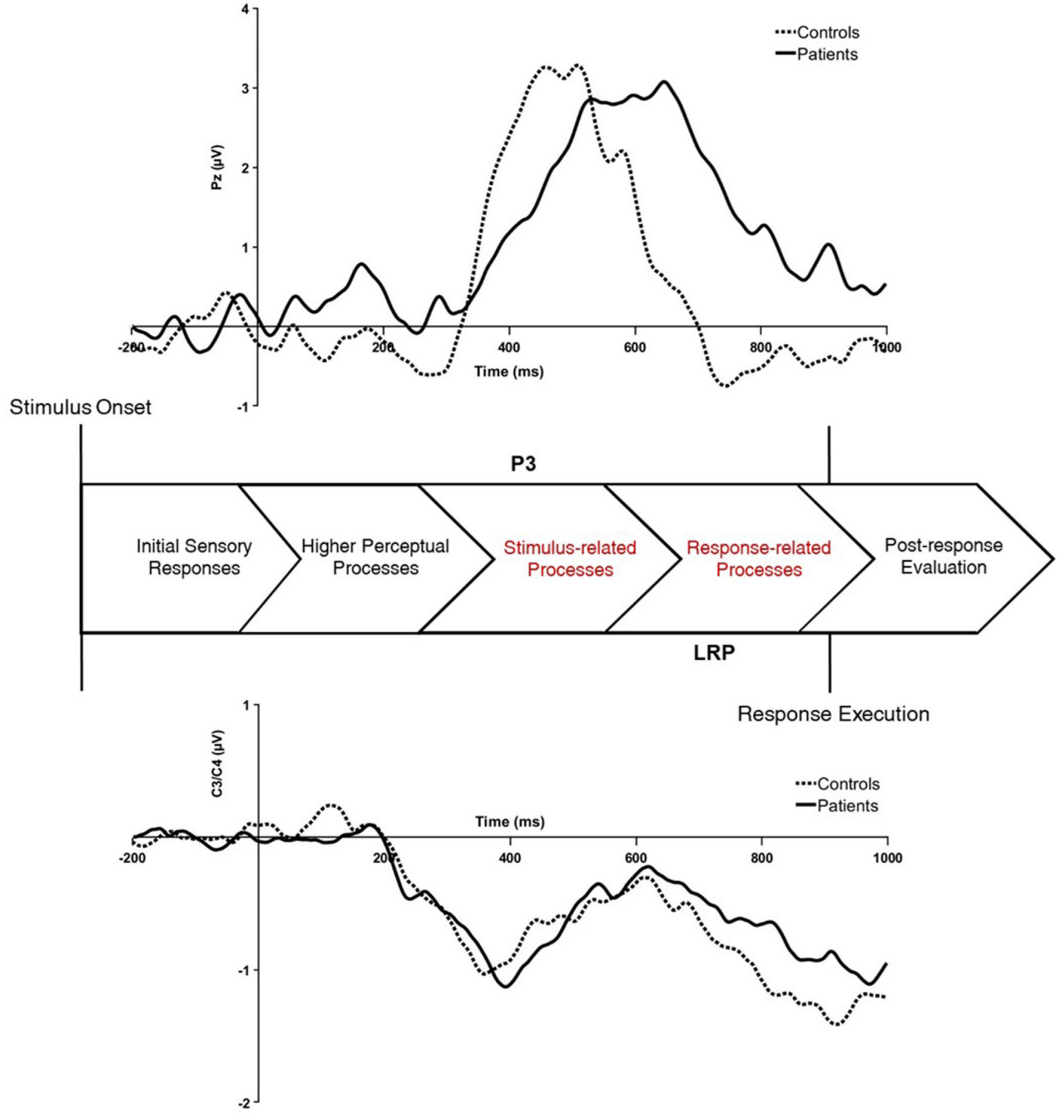
Stimulus-locked ERP difference waveforms for the rare-minus-frequent P3 difference waveform **(top)** and contralateral-minus-ipsilateral LRP difference waveform **(bottom)** for healthy controls and patients with first-episode schizophrenia. The P3 difference waveform was extracted from the midline parietal site (Pz) and the LRP was extracted from lateral central sites (C3/C4). μV, microvolts; ms, milliseconds.

**Figure 2:**
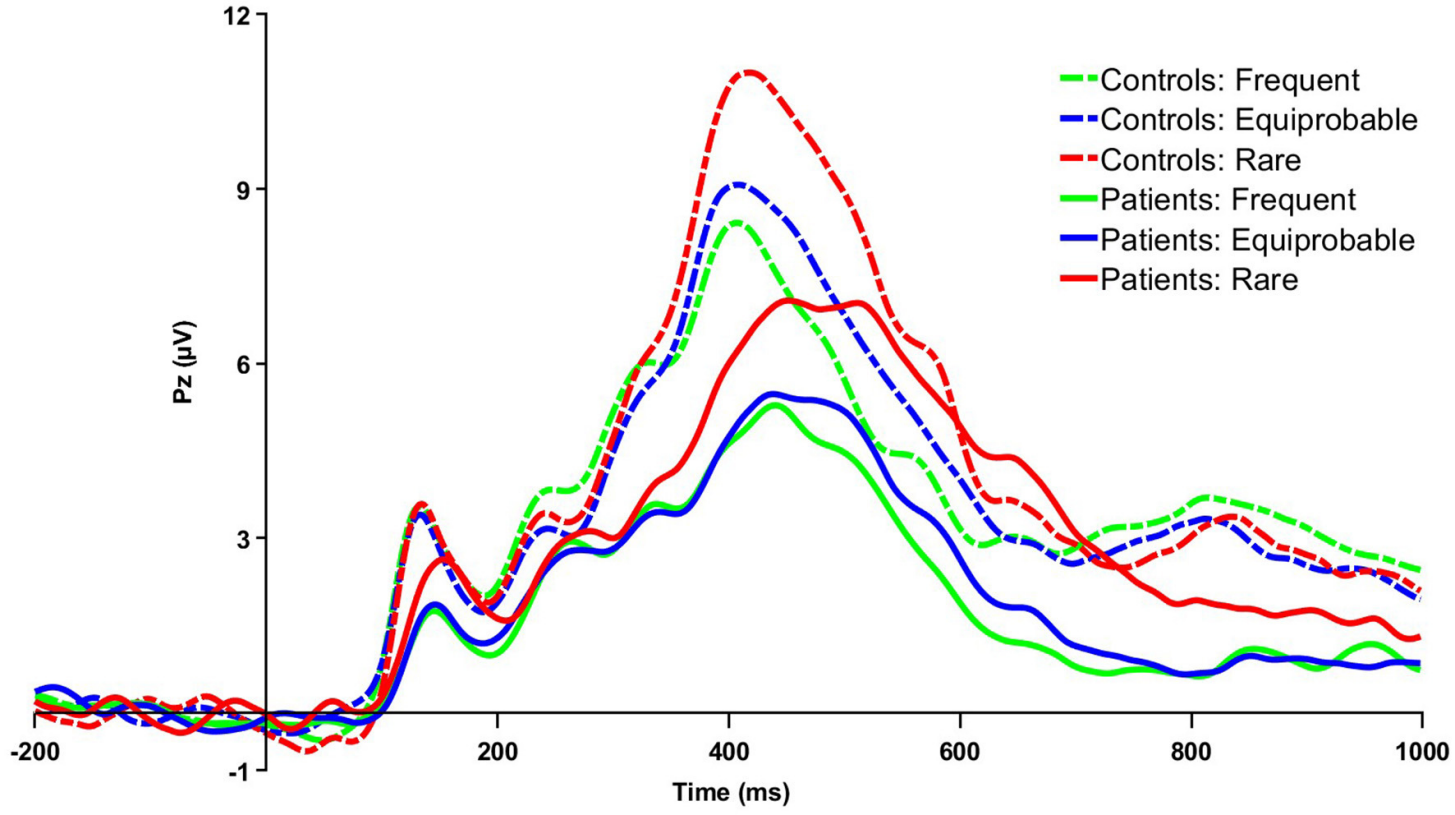
Grand averaged parent P3 waveforms for patients and controls for frequent, equiprobable, and rare trials at the midline parietal electrode site (Pz). μV, microvolts; ms, milliseconds.

One patient was excluded from the P3 analyses because more than 50% of the trials were rejected due to electrophysiological artifacts. However, their LRP data remained intact and were therefore included in the LRP analyses. One patient and one healthy control were excluded from LRP analysis due to amplitudes that prevented extraction of 50% peak onset latency. However, their P3 data were included in the P3 analyses. Primary difference waveforms are shown in Figure 1 and related analyses are shown in Table 4. LRP analyses are shown in Table 5. Parent P3 waveforms by group and probability are shown in Figure 2 and related analyses are shown in Table 6.

**Table 4:** Descriptive data for P3 and LRP difference waveform component measures by group.

**ERP measure**	**Patients**	**Controls**	**Statistics**		
			***t***	***p***	***g***
P3diff amplitude (μV)	1.99 (1.88)	2.13 (9.07)	*t*_(17.63)_ = 0.24	0.81	0.09
P3diff latency (ms)	538.00 (29.27)	497.57 (32.11)	*t*_(25)_ = 3.41	< 0.01	1.27
LRP amplitude (μV)	−0.63 (0.82)	−0.67 (0.63)	*t*_(25)_ = 0.53	0.48	0.02
LRP latency (ms)	354.21 (62.83)	334.46 (42.19)	*t*_(25)_ = 1.83	< 0.05	0.69

Amplitude and latency values are displayed as M and (SD).

**Table 5:** Descriptive data for LRP difference waveform component measures by group and stimulus probability.

	**Patients**				**Controls**				**Statistics**		
									**Group**	**Probability**	**Group** × **probability**
**Dependent variable**	**Group**	**Freq**	**Rare**	**Equi**	**Group**	**Freq**	**Rare**	**Equi**			
Amplitude (μV)	−0.64 (6.42)	−0.48 (0.79)	−0.76 (0.64)	−0.67 (0.82)	−0.67 (6.66)	−0.51 (0.79)	−0.67 (0.64)	−0.847 (0.82)	*F*_(1, 26)_ = 0.21; *p* = 0.89; *η^2^p* < 0.01	*F*_(1.6, 41.5)_ = 3.56; *p* = 0.05; *η^2^p* = 0.12	*F*_(1.6, 41.5)_ = 0.79 *p* = 0.89 *η^2^p* < 0.01
Latency (ms)	354.2 (64.3)	335.08 (77.47)	383.95 (65.16)	343.69 (99.59)	334.46 (66.78)	322.62 (77.47)	338.31 (65.16)	342.46 (99.54)	*F*_(1, 24)_ = 1.19; *p* = 0.08; *η^2^p* = 0.12	*F*_(2, 48)_ = 1.76*; p* = 0.18*; η^2^p* = 0.07	*F*_(2, 48)_ = 0.89 *p* = 0.42 *η^2^p* = 0.04

Amplitude and latency values are displayed as M and (SD).

**Table 6:** Descriptive data for parent P3 waveform component measures by group and stimulus probability.

	**Patients**				**Controls**				**Statistics**		
									**Group**	**Probability**	**Group** × **probability**
**Dependent variable**	**Group**	**Freq**	**Rare**	**Equi**	**Group**	**Freq**	**Rare**	**Equi**	**df** = **1,25**	**df** = **2,50**	**df** = **2,50**
Amplitude (μV)	3.94 (2.74)	3.23 (2.51)	5.09 (3.11)	3.51 (2.83)	6.06 (2.74)	5.26 (2.51)	6.99 (3.12)	5.93 (2.83)	*F* = 4.02; *p* = 0.06; *η^2^p* = 0.14	*F* = 31.95; *p < * 0.001; *η^2^p* = 0.56	*F* = 0.66 *p* = 0.52 *η^2^p* = 0.03
Latency (ms)	475.1 (22.1)	456.77 (25.95)	493.23 (17.90)	475.39 (15.02)	453.33 (22.17)	444.71 (25.95)	458.71 (27.45)	456.57 (30.01)	*F* = 6.52; *p* = 0.02; *η^2^p* = 0.21	*F* = 27.11; *p < * 0.001; *η^2^p* = 0.52	*F* = 5.57 *p* = 0.01 *η^2^p* = 0.18

Amplitude and latency values are displayed as M and (SD).

#### 3.3.1 P3 difference and parent waveforms

Using the P3 difference wave, patients had longer P3 latency relative to controls *t*_(25)_ = 3.41, *p* < 0.01, *g* = 1.27. Although the amplitude of the rare-frequent difference wave did not significantly differ between groups, the amplitude of the parent P3 waveforms at all three probability levels was lower for patients relative to controls. The main effect of group was marginally significant, indicating patients had blunted parent P3 waveform amplitudes relative to controls, *F*_(1, 25)_ = 4.02, *p* = 0.06, η^2^p = 0.14. There was also a significant main effect of probability, *F*_(2, 50)_ = 31.95, *p* < 0.001, η^2^p = 0.56, with larger parent P3 amplitudes to rare relative to equiprobable and frequent trials. There was no significant group x probability interaction.

The 2x3 ANOVA on parent P3 waveform latency revealed a significant main effect of group *F*_(1, 25)_ = 6.52, *p* = 0.02, η^2^p = 0.21, with longer parent P3 latency for patients relative to healthy controls. There was also a main effect for probability *F*_(2, 50)_ = 27.11, *p* < 0.001, η^2^p = 0.52, indicating longer P3 latency for rare relative to equiprobable and frequent stimuli trials, as well as for equiprobable relative to frequent stimuli trials, *p'*s < 0.05. There was a significant interaction between group and probability, *F*_(2, 50)_ = 5.57, *p* =0.01, η^2^p = 0.18, with longer rare and equiprobable parent P3 latencies for patients relative to healthy controls.

#### 3.3.2 LRP

All analyses were conducted and reported using the LRP difference waveform. Patients had longer LRP latency relative to healthy controls *t*_(25)_ = 1.83, *p* < 0.05, *g* = 0.69, despite comparable amplitudes between the two groups. The 2 × 3 repeated measures ANOVA on LRP amplitude violated sphericity, and thus the Greenhouse-Geisser correction was used. A significant main effect of probability, *F*_(1.60, 41.48)_ = 3.56, *p* =0.05, η^2^p =0.12, revealed marginally larger amplitudes for rare relative to frequent trials, *p* = 0.051. No significant main effect of group or an interaction was observed for LRP amplitude. In terms of LRP latency, there was a significant main effect of group that was marginally significant, *F*_(1, 24)_ = 1.19, *p* =0.08, η^2^p = 0.12. This showed longer LRP latency values for patients relative to controls. There were no significant main effects or interaction by probability.

### 3.4 Correlations

See Tables 7, 8 for a full breakdown of the correlational analyses. Across the entire sample, there was a moderate inverse relationship between aerobic fitness and BMI, *r*_(24)_ = −0.37, *p* < 0.05. Physical activity level was also positively related to aerobic fitness, *r*_(24)_ = 0.51, *p* < 0.01.

**Table 7:** Bivariate correlations between physical health measures, behavioral performance, and ERP component measures in first-episode schizophrenia patients.

**Variables**	**1**	**2**	**3**	**4**	**5**	**6**	**7**	**8**	**9**	**10**	**11**
1. BMI											
2. Physical activity	−0.32										
3. VO_2_	−0.47	0.48									
4. Accuracy	−0.08	0.38	0.44								
5. Median RT	0.29	−0.27	−0.16	−0.36							
6. LRP amplitude	−0.31	0.20	0.22	−0.32	−0.35						
7. LRP latency	0.34	0.01	0.20	0.30	−0.03	−0.12					
8. P3diff amplitude	−0.17	0.58 ^†^	0.07	0.23	−0.29	0.24	0.35				
9. P3diff latency	−0.38	0.18	0.14	−0.02	0.33	−0.18	0.01	0.35			
10. Parent P3 amplitude	−0.49^†^	0.21	−0.04	0.28	−0.70^**^	0.37	0.01	0.51^†^	0.03		
11. Parent P3 latency	−0.08	0.45	−0.08	0.28	−0.39	−0.20	0.12	0.29	−0.27	0.34	

BMI, body mass index.

^†^p < 0.1; ^**^p < 0.01.

**Table 8:** Bivariate correlations between physical health measures, behavioral performance, and ERP component measures in nonpsychiatric healthy controls.

**Variables**	**1**	**2**	**3**	**4**	**5**	**6**	**7**	**8**	**9**	**10**	**11**
1. BMI											
2. Physical activity	−0.09										
3. VO_2_	−0.18	0.44									
4. Accuracy	−0.07	−0.54^*^	−0.17								
5. Median RT	0.42	0.09	0.18	−0.10							
6. LRP amplitude	−0.02	0.07	−0.17	0.10	−0.43						
7. LRP latency	0.12	0.44	0.23	−0.29	0.62^*^	−0.17					
8. P3diff amplitude	−0.37	−0.31	−0.17	0.15	−0.44	0.09	−0.13				
9. P3diff latency	−0.20	0.35	−0.02	−0.52^†^	−0.21	−0.06	−0.32	−0.25			
10. Parent P3 amplitude	0.41	−0.05	−0.35	−0.11	0.07	−0.16	0.20	0.21	−0.12		
11. Parent P3 latency	0.18	0.38	0.13	−0.64^*^	0.33	−0.44	0.35	−0.37	0.28	0.44	

BMI, body mass index.

^†^p < 0.1; ^*^p < 0.05.

#### 3.4.1 Behavioral performance and ERP measures

Several associations between behavioral performance and ERP measures were observed across the entire sample that approached significance. Reaction time was negatively associated with parent P3 waveform amplitude, *r*_(25)_ = −0.35, *p* = 0.07, and LRP amplitude, *r*_(25)_ = −0.37, *p* = 0.06, and accuracy was negatively associated with P3 difference waveform latency *r*_(25)_ = −0.35, *p* = 0.08. In patients, reaction time was negatively significantly associated with parent P3 waveform amplitude *r*_(11)_ = −0.70, *p* < 0.01. In nonpsychiatric controls, reaction time was positively associated with LRP latency, *r*_(12)_ = 0.62, *p* < 0.05. Additionally, accuracy was negatively associated with parent P3 waveform latency, *r*_(12)_ = −0.64, *p* = 0.01, and was marginally associated with P3 difference waveform latency *r*_(12)_ = −0.52, *p* = 0.06.

#### 3.4.2 Physical health and cognition outcomes

For physical health-related attributes across the entire sample, BMI was positively associated with reaction time, *r*_(27)_ = 0.39, *p* < 0.05, and marginally albeit nonsignificantly associated with LRP latency, *r*_(25)_ =0.32, *p* = 0.10. In patients, physical activity was marginally associated with P3 difference waveform amplitude, *r*_(9)_ =0.58, *p* = 0.06, and parent P3 waveform amplitude was marginally associated with BMI *r*_(11)_ = −0.49, *p* = 0.09. In non-patient controls, accuracy was negatively associated with physical activity, *r*_(12)_ = −0.54, *p* < 0.05.

## 4 Discussion

The purpose of this study was to examine early stimulus perception and categorization and later response selection and preparation processing stages in young adult first-episode schizophrenia patients using the P3 and LRP difference waveforms, and to explore their association with physical activity, cardiorespiratory fitness, and BMI. Difference waveforms were used to isolate cognitive processes involved in stimulus perception and categorization (P3 rare—frequent difference waveform) and subsequent response selection and preparation (LRP contralateral—ipsilateral difference waveform) (Luck et al., 2009). Cognitive impairment in patients was indicated by behavioral performance and neurocognitive measures obtained during a simple stimulus discrimination oddball task. Relative to non-psychiatric controls, patients had slower reaction time and reduced accuracy to the task, and longer P3 and LRP component latencies. Patients also reported significantly lower physical activity levels than controls. Early stimulus-related stages of information processing reflected in the amplitude of the P3 difference wave was associated with physical activity in patients, although this correlation did not reach significance. In general, these findings corroborate previous studies indicating cognitive impairment early in the first-episode phase of schizophrenia (Addington and Addington, 2002; McCleery et al., 2014) and suggest impaired metrics of physical health in first-episode schizophrenia patients, with physical activity potentially serving as a behavioral treatment target for enhancing neurocognitive function in this patient population.

In contrast to a previous study (Luck et al., 2009), first-episode patients exhibited delayed information processing across both stimulus categorization and response selection processes relative to healthy controls, as indicated by longer P3 and LRP difference waveform component latencies. Luck et al. (2009) found attenuated and delayed LRP components in schizophrenia patients (mean age = 47 years) who were all receiving antipsychotic medication relative to controls despite comparable P3 difference waveforms, suggesting that the delayed reaction times in adults with schizophrenia are primarily a consequence of response selection and preparation processes. Younger first-episode schizophrenia patients in the present study exhibited similar delays in response-related processes, as indicated by longer LRP latency relative to non-patients. However, in contrast to the previous findings from Luck et al. (2009), the latency of the P3 difference waveform was longer among patients in this study, suggesting delays across stages of information processing, including stimulus perception and categorization (P3) as well as response selection and preparation (LRP). It is possible that differences in our findings and those of Luck et al. (2009) are merely due to the cognitive heterogeneity between individual patients with schizophrenia (Joyce and Roiser, 2007). Cognitive impairment in schizophrenia can be influenced by a number of etiological and environmental factors, including genetics, early life experiences, substance use, and physical health status (McCutcheon et al., 2023). It is also possible that group level differences in our study and those from Luck et al. (2009) are a product of the comparison group. The P3 latencies (reported as M, SD) in the Luck et al. (2009) study at the midline parietal electrode site for patients was (572.72, 12.01) and for controls was (567.67, 15.06). In contrast, these values in the current study were (538, 29.27) for patients and (497.57, 32.11) for controls. Therefore, the between group difference in P3 latency may be due to the shorter P3 latency found for the nonpsychiatric comparison controls. The nonpsychiatric control group in this study was relatively young and healthy, which may be linked to improved cognition as reflected by the P3 wave (Kao et al., 2020). Together, these findings suggest that information processing deficits are evident early in the course of schizophrenia and initially span both stimulus- and response-related information processing stages. Future studies are warranted to tease apart the temporal stages of cognitive slowing in schizophrenia, as well as to examine individual differences in the trajectory of cognitive slowing.

### 4.1 Physical health and neurocognition

The current findings suggest modest associations between physical health metrics and stimulus- and response-related cognitive processes. Across the entire sample, higher BMI was associated with slower reaction times, which corroborates previous reports of cognitive impairment among individuals with higher BMI (Burkhalter and Hillman, 2011; Prickett et al., 2015; Dye et al., 2017). BMI has been shown to be consistently higher in schizophrenia patients (Annamalai et al., 2017), which may be impacted by antipsychotic medication (Wirshing, 2004; Manu et al., 2015) and lifestyle behaviors (Strassnig et al., 2012; Harvey and Strassnig, 2019). In patients in this study, BMI had expected negative associations with P3 and LRP amplitudes, as well as a positive correlation with LRP latency; however, these correlations were not statistically significant. A systematic review and meta-analysis of 13 studies involving 2,800 individuals with schizophrenia found global cognitive deficits were larger in individuals with (vs without) metabolic syndrome (Hagi et al., 2021). However, when parsing the components of metabolic syndrome, diabetes and hypertension were associated with significant cognitive impairment, while obesity was not. Body composition, weight gain and obesity remain important health factors in schizophrenia although their impact on neurocognitive function may be complex and intertwined with related metabolic and lifestyle factors such as hypertension, diabetes, and physical inactivity.

First-episode schizophrenia patients reported lower physical activity levels than their age-matched nonpsychiatric counterparts. For patients, lower physical activity was also associated with an attenuated P3 difference waveform amplitude (*p* = 0.06), although this correlation did not reach conventional levels of significance. Attenuated P3 difference waveform amplitude has been interpreted within an aging-cognition framework (Brush et al., 2020) as age-related cognitive decline (Salthouse, 2000; Salthouse and Ferrer-Caja, 2003; Polich, 2012). A blunted P3 difference waveform may indicate less contextual updating when presented with rare stimuli (i.e., impaired stimulus perception or categorization) in oddball paradigms (Donchin and Coles, 1988; Polich, 2007; Kao et al., 2020). In-line with this interpretation, deficits in early stimulus-related stages of information processing may be largest in physically inactive first-episode schizophrenia patients. Previous systematic reviews have shown that individuals with schizophrenia engage in significantly less physical activity (Stubbs et al., 2016) and an increasing number of clinical trials are examining the influence of increasing physical activity on cognition in schizophrenia (e.g., Nuechterlein et al., 2016). Cognitive impairment in schizophrenia represents one of the main obstacles to clinical and functional recovery and has proven challenging to address (Harvey et al., 2022). Physical activity appears to be a promising behavioral treatment intervention with the potential to ameliorate this insidious feature of schizophrenia.

Despite reports of associations between cardiorespiratory fitness and neurocognition in schizophrenia patients (Kimhy et al., 2014), we did not observe relations between cardiorespiratory fitness and information processing. One explanation for this involves the dependent measure of cognition. Reported associations between cardiorespiratory fitness and neurocognition come from end-state behavioral measures obtained from the MATRICS Consensus Cognitive Battery (Kimhy et al., 2014) whereas the present null findings were obtained from ERP components that are specific to stages of information processing during a simple visual stimulus discrimination task. The difference in findings highlights critical differences in the assessment of cognition and the ecological validity of assessments used in patient populations. Additionally, the present findings highlight differences in physical health between first-episode patients relative to those in more progressed stages of mental illness. Cardiorespiratory fitness was lower in schizophrenia patients in Kimhy et al. (2014) relative to first-episode patients in the present study (M = 21.5 mL/kg/min, SD = 6.5 vs. M = 33.7 mL/kg/min, SD = 7.7), and cardiorespiratory fitness did not differ between first-episode schizophrenia patients and non-patients (M = 33.7 mL/kg/min, SD = 7.7 vs. M = 38.9 mL/kg/min, SD = 9.2) in the present study. Together, these findings suggest the value of early intervention to preserve both physical health, cognition and functionality in this patient population. Additionally, it remains to be determined whether lifestyle physical activity or exercise that results in improvements in cardiorespiratory fitness are necessary to enhance cognition, symptoms, and physical function in schizophrenia patients across the prodromal, active, or residual phases of the disease.

## 5 Limitations

Our study has several limitations. Participants included young adults with first-episode schizophrenia who were recruited for an exercise intervention targeting neurocognition and age-matched psychiatrically healthy control participants were selected from a previous cross-sectional study (Brush et al., 2020). This selection process may have led to a relative over-sampling of higher functioning patients who were willing to participate in exercise, which would tend to decrease differences between groups. However, the comparison group was a younger healthy sample relative to previous cross-sectional studies in schizophrenia, which could have led to the significant group differences in the cognitive outcomes, particularly for the P3 latency difference noted in this study relative to the Luck et al. (2009) study. The cross-sectional design prevents us from drawing any causal interpretations regarding relationships between physical health characteristics and neurocognition. Furthermore, the patients in this study also were more likely to be cognitively intact and able to travel outside the home, potentially impacting both the cognitive and physical health measures. An unexpected finding was that physical activity was found to be negatively correlated with response accuracy in the nonpsychiatric controls. When looking at the controls separately, the higher physical activity group (based on a median split) had accuracy rates of 0.96 (0.03) while the lower active split had rates of 0.98 (0.02). It is likely that this spurious finding was driven by a ceiling effect of the task, since previous studies using behavioral performance measures have shown benefits from physical activity (Haverkamp et al., 2020).

The small sample size in this study likely influenced the lack of significance between some of our physical health metrics and ERP outcomes. The exercise intervention study had to be halted due to the COVID-19 shutdown, limiting our ability to recruit additional first-episode schizophrenia patients. Also related to our sample, we had trouble obtaining clean EEG data from several patients. Patients had fewer clean EEG trials relative to non-patients due to excessive movement-related artifact, which in one case resulted in the discarding of more than 50% of trials. Future studies should examine trajectories of association between physical health metrics and cognition across the course of the illness, from the premorbid and prodromal phases to first episode and chronic schizophrenia. Finally, many confounding factors may influence both physical and cognitive health in schizophrenia, including smoking, substance abuse, obesity, and metabolic syndrome (Harvey et al., 2022).

## 6 Conclusion

In conclusion, the current findings suggest cognitive impairment in first-episode schizophrenia at the behavioral and neurocognitive level, and that information processing may be associated with key aspects of physical health. Patients exhibited deficits in behavioral task performance (i.e., accuracy and reaction time) and neurocognition (i.e., longer P3 and LRP difference waveform latencies) relative to age-matched controls. Patients also reported significantly lower physical activity levels, which were marginally associated with lower P3 amplitude. These findings highlight that cognitive impairment spans both stimulus- and response-related stages of information processing, can manifest by first-episode stages of disease progression, and may be associated with physical health. Because cognitive impairment is central to functional limitations experienced by people with schizophrenia, it may be a worthwhile component of the disease to target through interventions. While the present associations between information processing and physical health did not reach statistical significance, they suggest that in addition to existing treatments, future interventions should consider targeting modifiable aspects of physical health, such as physical activity, in order to preserve functionality. Although, sufficiently powered longitudinal studies are necessary to assess whether physical health improvements confer cognitive benefits in schizophrenia, the present suggests the potential utility of physical health for cognitive function during first-episode schizophrenia.

## Data availability statement

The raw data supporting the conclusions of this article will be made available by the authors, without undue reservation.

## Ethics statement

The studies involving humans were approved by Rutgers Institutional Review Board. The studies were conducted in accordance with the local legislation and institutional requirements. The participants provided their written informed consent to participate in this study.

## Author contributions

LP: Writing—original draft, Writing—review & editing. AU: Writing—original draft, Writing—review & editing, Conceptualization, Methodology. HP: Writing—original draft, Writing—review & editing. JB: Writing—original draft, Writing—review & editing. AS: Writing—original draft, Writing—review & editing. AD: Writing—original draft, Writing—review & editing. BA: Writing—original draft, Writing—review & editing.
